# Amniotic-Fluid–Derived Mesenchymal Stem Cells Overexpressing Interleukin-1 Receptor Antagonist Improve Fulminant Hepatic Failure

**DOI:** 10.1371/journal.pone.0041392

**Published:** 2012-07-23

**Authors:** Yu-Bao Zheng, Xiao-Hong Zhang, Zhan-Lian Huang, Chao-Shuang Lin, Jing Lai, Yu-Rong Gu, Bin-Liang Lin, Dong-Ying Xie, Shi-Bin Xie, Liang Peng, Zhi-Liang Gao

**Affiliations:** 1 Department of Infectious Diseases, The Third Affiliated Hospital, Sun Yat-sen University, Guangzhou City, People’s Republic of China; 2 Key Laboratory of Tropical Disease Control, Sun Yat-Sen University, Ministry of Education, Gangding, Guangzhou City, People’s Republic of China; Leibniz Institute of Age Research - Fritz Lipmann Institute, Germany

## Abstract

Uncontrolled hepatic immunoactivation is regarded as the primary pathological mechanism of fulminant hepatic failure (FHF). The major acute-phase mediators associated with FHF, including IL-1β, IL-6, and TNF-α, impair the regeneration of liver cells and stem cell grafts. Amniotic-fluid–derived mesenchymal stem cells (AF-MSCs) have the capacity, under specific conditions, to differentiate into hepatocytes. Interleukin-1–receptor antagonist (IL-1Ra) plays an anti-inflammatory and anti-apoptotic role in acute and chronic inflammation, and has been used in many experimental and clinical applications. In the present study, we implanted IL-1Ra–expressing AF-MSCs into injured liver via the portal vein, using D-galactosamine–induced FHF in a rat model. IL-1Ra expression, hepatic injury, liver regeneration, cytokines (IL-1β, IL-6), and animal survival were assessed after cell transplantation. Our results showed that AF-MSCs over-expressing IL-1Ra prevented liver failure and reduced mortality in rats with FHF. These animals also exhibited improved liver function and increased survival rates after injection with these cells. Using green fluorescent protein as a marker, we demonstrated that the engrafted cells and their progeny were incorporated into injured livers and produced albumin. This study suggests that AF-MSCs genetically modified to over-express IL-1Ra can be implanted into the injured liver to provide a novel therapeutic approach to the treatment of FHF.

## Introduction

Fulminant hepatic failure is a serious clinical condition that is associated with a high mortality rate. Orthotopic liver transplantation is the treatment of choice for FHF and end-stage liver disease [1]. However, liver transplantation has limitations, primarily due to a lack of readily available donors. Bioartificial liver devices could ideally serve as a bridge to transplantation or liver regeneration in patients with FHF. According to recent clinical results, Bioartificial liver systems containing animal hepatocytes have proven to be safe, but immunological rejection and zoonosis are still major problems [2].

A recent study has reported that stem cells derived from second-trimester amniocentesis were pluripotent, with the capacity to differentiate into multiple lineages, including representatives of all three embryonic germ layers [3], [4]. And our previous study [5] showed that AF-MSCs exhibited a greater capacity for cell proliferation, self-renewal, and hepatic differentiation than do bone marrow-derived mesenchymal stem cells. AF-MSCs may thus provide an ethically uncontroversial and easily accessible source of human hepatocytes for future clinical applications. And mesenchymal stem cells have been of interest because of their accessibility and amenability to transfection with exogenous genes [6].

Recent studies have shown that FHF is an inflammatory disease; this conclusion is supported clinically by elevated serum levels of immuno-inflammatory cytokines, including IL-1, TNF, IL-6 [7], and IL-8 [8]. Uncontrolled hepatic immunoactivation is regarded as the primary pathological mechanism of FHF [9]. Previous studies have shown that the cytotoxic effects of inflammatory cytokines inhibit both liver regeneration and the differentiation of engrafted stem cells into hepatocytes [10]. IL-1 plays an important role in initiating the cascade of events involved in immuno-inflammatory responses, exerting its effects on a wide variety of cells and often leading to tissue destruction, predominantly in the liver [11]. Therefore, both stem cell-based therapies and the blockade of inflammatory cytokines may be beneficial in the treatment of liver injury.

IL-1Ra is a naturally occurring cytokine and a member of the IL-1 family whose only known function is to prevent a biological response to IL-1 by competing for its receptor. The balance between endogenous IL-1 and IL-1Ra in vivo is an important determinant of the host response to infection [12]. Recent clinical findings also suggest that an imbalance between IL-1 and IL-1Ra in tissues may contribute to the pathogenesis and activation of chronic active hepatitis C [13]. Moreover IL-1Ra not only prevents a biologic response to IL-1 but also has been shown to attenuate IL-1– induced apoptosis [14]. IL-1Ra is thus considered to have hepato-protective effects [7] and to play an anti-inflammatory role in acute and chronic inflammation [12].

Based on this concept, we tested whether transfusion of IL-1Ra-expressing AF-MSCs could protect damaged livers in a rat FHF model by suppressing excessive immunoinflammatory responses and promote regeneration after cell transplantation.

## Results

### Characteristics and Human IL-1Ra Gene Transfer in AF-MSCs

**Figure 1:**
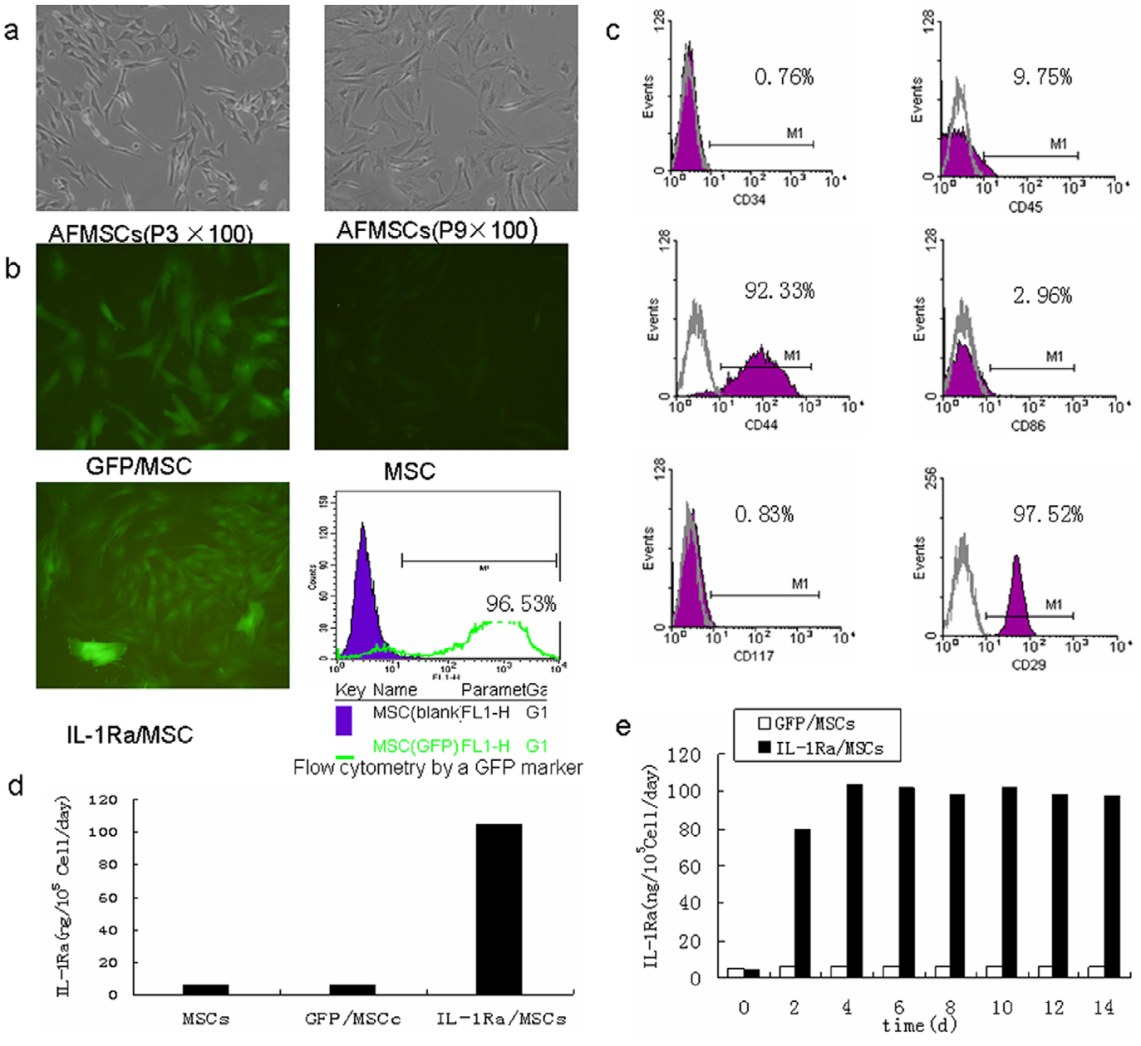
Characterization of amniotic-fluid–derived mesenchymal stem cells (AF-MSCs). (a) Morphological characterization of AF-MSCs. (b) MSCs were transduced with lentivirus green fluorescence protein (LV-GFP) or LV- human Interleukin-1–receptor antagonist (LV-hIL-1Ra) and the transduction efficiency of AF-MSCs was detected by flow cytometry using a GFP marker. (c) Fluorescence-activated cell sorting analysis of rat AF-MSCs. Percentage in the panels represents mean fluorescence intensity of the cells expressing each marker. (d) hIL-1Ra concentrations in the supernatants of MSCs, GFP/MSCs, and hIL-1Ra/MSCs. (e) LV-mediated expression of hIL-1Ra by MSCs over time.

AF-MSCs retained a fibroblastic morphology after repeated passages (Figure 1a), and their immunophenotypical characterization was confirmed by flow cytometry. Over 90% of the isolated AF-MSCs expressed CD29 and CD44, but not CD34, CD45, CD86 or CD117 (Figure 1c). These results are consistent with well-established markers of bone-marrow–derived MSCs(BM-MSCs) [18], [19].

AF-MSCs transduced with lentiviral vectors(Lv)-IL-1Ra were designated as IL-1Ra/MSCs. 96.53% of IL-1Ra/MSCs were positive for GFP on day 3 after transduction(Figure 1b). Peak concentrations of hIL-1Ra(10^4^ ng/10^5^ cells/day) were detected on day 4 after transduction (Figure 1d) in the cultured supernatants. Lentivirus-mediated stable expression of IL-1Ra by MSCs was maintained for at least 14 days in vitro (Figure 1e).

### IL-1Ra/MSCs Decrease FHF Mortality Rates

**Figure 2:**
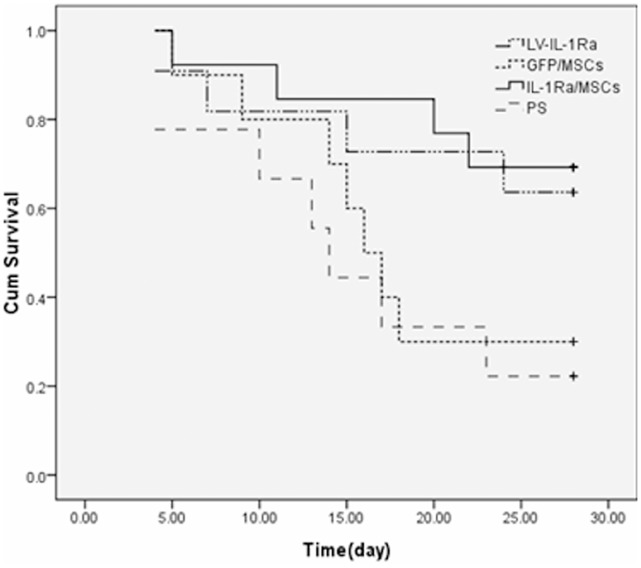
Kaplan-Meier plot showing the pattern of survival of fulminant hepatic failure (n = 15/group) after various treatments. LV-IL-1Ra, Lentiviral-Interleukin-1 receptor antagonist; MSCs, mesenchymal stem cells; PS, physiological saline; GFP, green fluorescence protein.

There was no significant difference between the survival rates in any of the rodent groups until 96 hours after cell transplantation. However, the survival rates of the lentiviral vectors -IL-1Ra(LV/IL-1Ra) and MSC transduced with lentiviral vectors-IL-1Ra (IL-1Ra/MSC) groups were higher than those of the physiological saline (PS) and MSC transduced with lentiviral vectors-GFP (GFP/MSC) groups after 96 hours of cell perfusion. The 21-day survival rates of rats in the PS, GFP/MSC, Lv/IL-1Ra, and IL-1Ra/MSC groups were 22.2%, 30.0%, 63.6%, and 69.2%, respectively (*P*<0.05; Figure 2). These results show that the survival rates of animals transplanted with IL-1Ra/MSCs or Lv/IL-1Ra are markedly higher than those of the PS and GFP/MSC groups.

### IL-1Ra/MSCs Improved Liver Function by Promoting Proliferation and Suppressing Apoptosis of Hepatocytes

**Figure 3:**
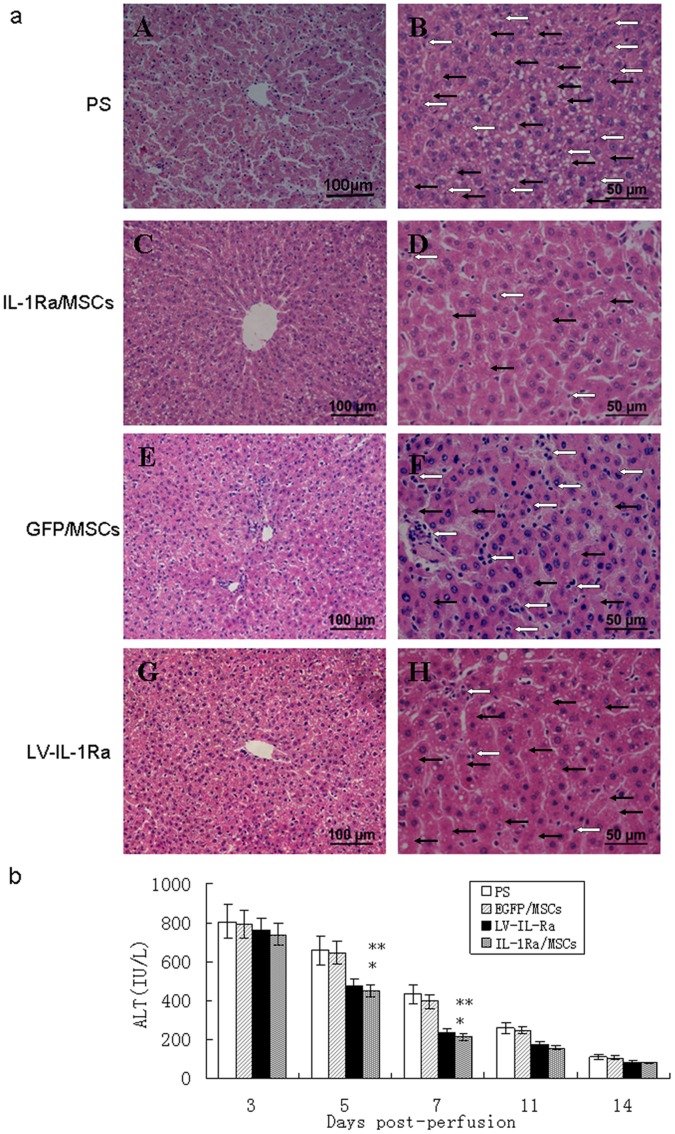
Hepatic injury in rat FHF was assessed by histological analyses and serum liver enzymes after cell perfusion: (a) Apparent structural disorder of liver lobules was detected in the physiological saline (PS) group and lentiviral-interleukin-1 receptor antagonist (LV-IL-1Ra) group (A,G). Increased infiltration of mononuclear cells(white arrow), vacuolar degeneration, and necrosis(black arrow) were observed in the PS group(B, F, H), Increased infiltration of mononuclear cells were observed in the green fluorescent protein (GFP)/MSC group(F), Increased vacuolar degeneration, and necrosis were observed in the LV-IL-1Ra group (H), as compared to the interleukin-1 receptor antagonist/mesenchymal stem cell (IL-1Ra/MSC) group (D). Original magnification ×200 (A, C, E, G), ×400 (B, D, F, H). (b) There were significantly lower alanine aminotransferase (ALT) levels in IL-1Ra/MSC rats than those in the PS and green fluorescent protein (GFP)/MSC groups by day 5 after cell transplantation. *P<0.05, when compared to the PS and GFP/MSC groups. **P>0.05 when compared to the lentiviral-interleukin-1 receptor antagonist (LV-IL-1Ra) group.

The degree of hepatic injury in our rat FHF model was assessed by histological analyses (Figure 3a). Histological manifestations of liver injury, including marked infiltration of mononuclear cells and destruction of the liver architecture in periportal areas, were first observed on day 5 after induction. Evident dilation of the sinusoids and some areas of necrosis were also seen. In IL-1Ra/MSCs, slight perivascular infiltration of mononuclear cells and small focal areas of hepatocyte necrosis were also detected (Figure 3a). Rats in the PS and IL-1Ra/MSCs groups showed histological injuries similar to those observed in the GFP/MSC and Lv/IL-1Ra groups, respectively.

Serum levels of ALT were measured on days 3, 5, 7, 11, and 14 after cell transplantation in order to evaluate the extent of hepatocellular lesions. On day 5, the IL-1Ra/MSC group had significantly lower levels of ALT (448.7±112.6 IU/L), than the PS group (657.3±147.4 IU/L) (*P<*0.05; n = 5). On day 7, the serum ALT levels of the IL-1Ra/MSC group (215.7±78.3 IU/L) were further decreased to half that of the PS group (435.8±122.7 IU/L) (*P<*0.05; n = 5). All of the rats with high ALT levels in the PS and GFP/MSC groups died between days 3 and 5. In contrast, the drawdown of ALT levels was detected in IL-1Ra/MSCs-transfused and Lv/IL-1Ra-infected rats despite a mild transient increase in ALT (Figure 3b).

**Figure 4:**
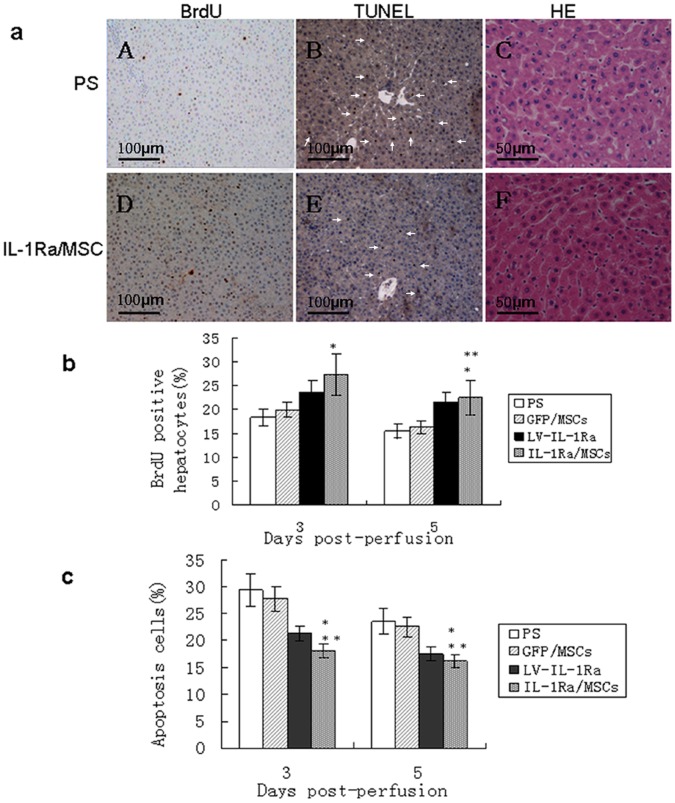
MSCs over-expressing IL-1Ra promote proliferation and suppress apoptosis of hepatocytes. (a) Immunohistochemical staining of bromodeoxyuridine (BrdU) incorporation and 2′-deoxyuridine 5′-triphosphate nick-end labeling (TUNEL) assay on days 3 and 5 after cell perfusion. Representative immunohistochemistry at 72 hours (A, D). (b) Graph of the average percentage of BrdU-positive nuclei at the indicated times after cell transplantation. TUNEL-positive cells on days 3 and 5 (B, E). (c) Graph of the average percentage of TUNEL-positive cells. Original magnification ×200 (A, B, D, E), ×400 (C, F). *Versus physiological saline (PS) and green fluorescent protein/mesenchymal stem cell (GFP/MSC) groups, *P<*0.05. **Versus lentiviral-interleukin-1 receptor antagonist (LV-IL-1Ra), *P>*0.05. H & E, hematoxylin and eosin.

In order to explore the mechanism of proliferation promoted by IL-1Ra in hepatocytes, immunohistochemical analyses were conducted using anti-BrdU antibodies. Seventy-two hours after cell transplantation, a significant increase in BrdU-positive hepatocytes was noted in the IL-1Ra/MSC group (27.3±1.23%) compared to the PS, GFP/MSC and LV-IL-1Ra groups (18.32±1.21%, 19.9±1.33% and 23.5±1.34%, respectively; *P<*0.05, n = 5). The fraction of labeled hepatocytes was significantly lower on day 5 in each of the groups (Figure 4b) and was not significantly different between the IL-1Ra/MSC (22.5±1.03%) group and the LV-IL-1Ra group (21.6±1.31%; *P*>0.05, n = 5). Concomitantly, more mitotic hepatocytes were observed in the IL-1Ra/MSC rats 5 days after cell transplantation (Figure 4a).

On day 5 after cell transplantation, a clearly lower percentage of apoptotic liver cells was noted in the IL-1Ra/MSCrats compared to the PS and GFP/MSC rats (18.3±1.5% vs 27.6±1.2% and 24.5±1.8%; *P<*0.05, n = 5). However, no significant difference in the percentage of apoptotic liver cells was observed between the IL-1Ra/MSC and LV-IL-1Ra rats (Figure 4a, c).

**Figure 5:**
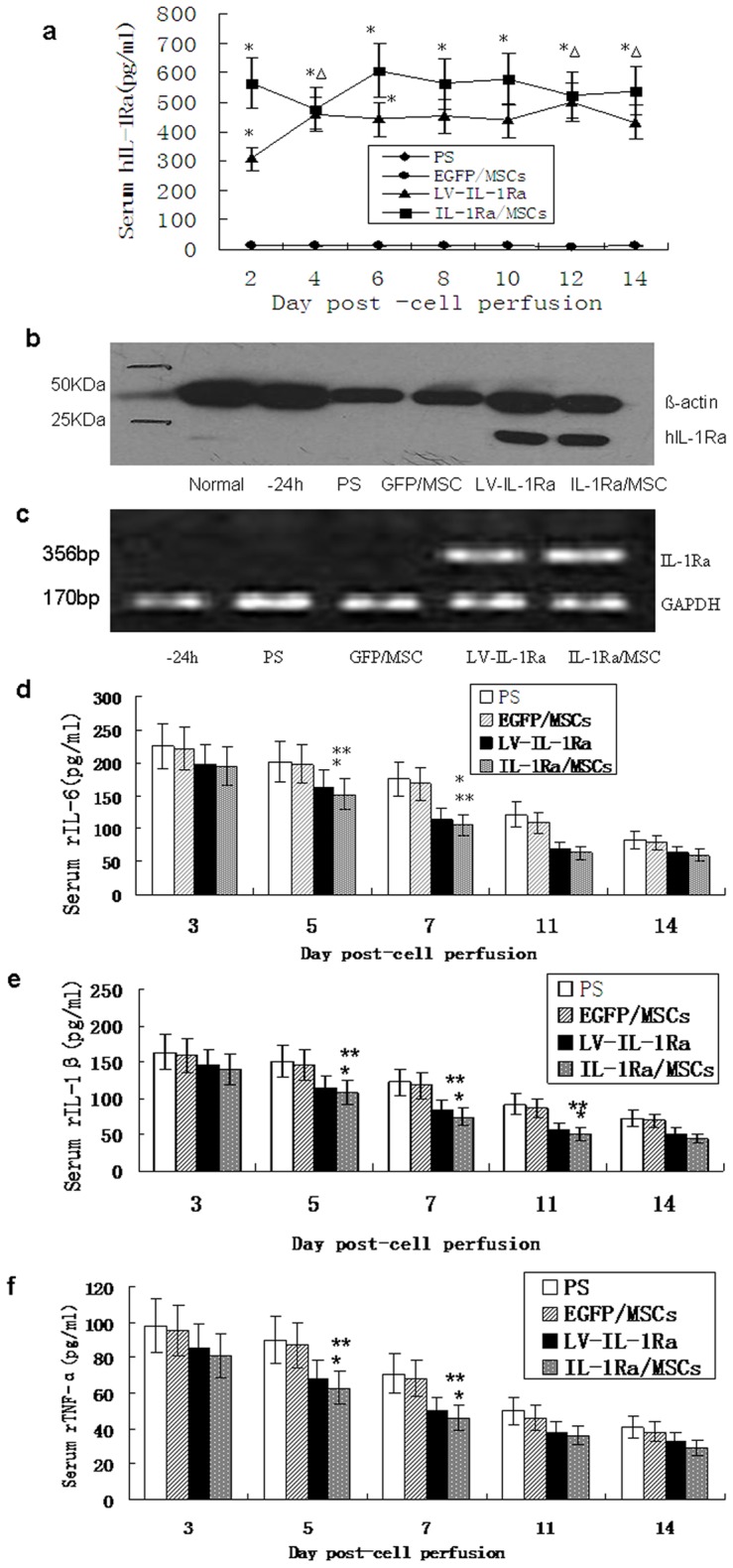
Expression of human interleukin-1 receptor antagonist (hIL-1Ra) in rats with FHF treated with IL-1Ra/mesenchymal stem cells (MSCs) and lentiviral-hIL-1Ra. (a) Serum hIL-1Ra concentrations were determined by enzyme-linked immunosorbent assay (ELISA). *P<0.05, when compared to the physiological saline (PS) and green fluorescent protein (GFP)/MSC groups; *^Δ^P>*0.05, when compared with lentiviral-interleukin-1 receptor antagonist (LV-IL-1Ra) groups, respectively. (b) Western blot analysis of hIL-1Ra protein before and after various treatments on day 14 after cell transplantation. (c) The hIL-1Ra messenger RNA (mRNA) levels in failing liver tissue of rats with FHF on day 2 after various treatments. The time-point of 24 hours prior to liver transplantation was the control. (d) Serum inflammatory cytokine IL-6 concentrations by enzyme-linked immunosorbent assay (ELISA) at various time points after cell perfusion. (e) Serum inflammatory cytokine IL-1β concentrations by ELISA at various time points after cell perfusion. (f) Serum inflammatory cytokine TNF-α concentrations by ELISA at various time points after cell perfusion. *P<0.05 when compared to the physiological saline (PS) and green fluorescent protein (GFP)/MSC groups; **P>0.05 when compared to LV-IL-1Ra group.

IL-1Ra/MSCs improved liver function by producing hIL-1Ra and down-regulating the inflammatory responses activated by interleukin-1 in vivo. In order to ascertain that hIL-1Ra is expressed in IL-1Ra/MSCs in vivo, we measured serum hIL-1Ra concentrations by ELISA (Figure 5a). hIL-1Ra levels were detectable in IL-1Ra/MSC and Lv/IL-1Ra rats for approximately 2 days after cell perfusion. The IL-1Ra/MSC rats showed peak serum levels of hIL-1Ra at approximately 1 week after cell perfusion, while the Lv/IL-1Ra rats showed peak levels on day 4. In both groups, serum hIL-1Ra was steadily expressed at peak levels by day 6 after cell transplantation. Notably, the expression levels of hIL-1Ra in IL-1Ra/MSC rats were significantly higher than that of Lv/IL-1Ra rats on days 6, 8, and 10 after cell transplantation (*P<*0.05, *P<*0.05 and *P<*0.05, respectively; n = 5). Levels of hIL-1Ra were almost undetectable in the PS and GFP/MSC rats.

The levels of IL-1Ra expression observed in IL-1Ra/MSC rats are consistent with the time-line for peak expression after lentiviral transduction in vitro (Figure 1e). In this study, the transduction of MSCs was carried out 5 days before transfusion into injured rat livers. Because the peak expression of lentivirus occurs after 96 hours of incubation in vitro, peak hIL-1Ra expression in Lv/IL-1Ra rats should occur on the fourth day after cell transplantation, which is 48 hours later than in the IL-1Ra/MSC group. These expression profiles are in agreement with our in vitro observations (Figure 1e).

In order to determine whether exogenous IL-1Ra is derived from in situ expression of the transgene in the liver, we investigated hIL-1Ra expression 24 hours before treatment, 72 hours after treatment, and 14 days after treatment with Lv/IL-1Ra or IL-1Ra/MSCs. As shown in Figure 5b, baseline levels of hIL-1Ra were not detectable. However, 14 days after cell transplantation, IL-1Ra protein levels were clearly higher in the IL-1Ra/MSC and Lv/IL-Ra rats than in the PS and GFP/MSC rats. No significant differences in IL-1Ra protein levels were observed between IL-1Ra/MSC and Lv/IL-1Ra rats at 96 hours after cell transplantation (data not shown).

IL-1Ra mRNA expression in IL-1Ra/MSC and Lv-IL-Ra rats was upregulated within 48 hours after cell transplantation and remained for up to two weeks but was undetectable in the PS and GFP/MSC rats during the same period of time. hIL-1Ra in mRNA levels was also not detected 24 hours before treatment (Figure 5c).

In order to determine whether IL-1Ra can attenuate inflammatory responses in FHF rats, we measured concentrations of the serum inflammatory cytokine IL-6, IL-1β and TNF-α using ELISA after cell perfusion (Figure 5d, e, f). In all groups, serum IL-6 gradually declined from a peak of 226 pg/ml to a minimal level of 62±6.7 pg/ml by day 14 after cell transplantation. Serum IL-6 levels in IL-1Ra/MSC rats were clearly lower than that of LV-IL-Ra rats by day 5 after cell perfusion (152±27.5 vs 201.5±33.4 pg/ml; n = 5, *P<*0.05). During the cell perfusion stage (day 14), serum IL-6 levels declined more rapidly in IL-Ra/MSC rats than in PS rats; in each of these groups, IL-6 levels declined in a manner similar to that observed for serum ALT levels. Moreover, in the IL-1Ra/MSC and Lv/IL-1Ra rats, there were significant reductions in serum levels of IL-6 compared to that of GFP/MSC rats (62±8.9 vs 121±18.3 pg/ml and 69±9.7 vs 121±18.3 pg/ml; n = 5, *P<*0.05 and *P<*0.05, respectively). Concomitant with serum IL-1Ra level changes, serum IL-1β and TNF-α levels gradually decreased until reaching the minimum level on day 14 after cell transplantation in all groups. Notably, the serum levels of IL-1β in IL-1Ra/MSC rats were significantly lower than in GFP/MSC rats on days 5, 7, and 11(108±16.02 vs 146±21.9 pg/ml; 75±11.25 vs 118±17.7 pg/ml; and 51±7.65 vs 87±13.05 pg/ml; n = 5, P<0.05 in all 3 comparisons). TNF-α levels declined in a manner similar to that observed for serum IL-6 and IL-1β levels. However, there were no differences in serum IL-6, IL-1β, and TNF-α levels between the 4 groups by day 11 after cell transplantation. Moreover, during 14 days after cell transplantation, no significant differences in the serum levels of inflammatory cytokines were observed between IL-1Ra/MSC and Lv/IL-1Ra rats.

### IL-1Ra Enhanced the Incorporation of MSCs into Injured Livers and Attenuated Intrahepatic Inflammatory Responses

**Figure 6:**
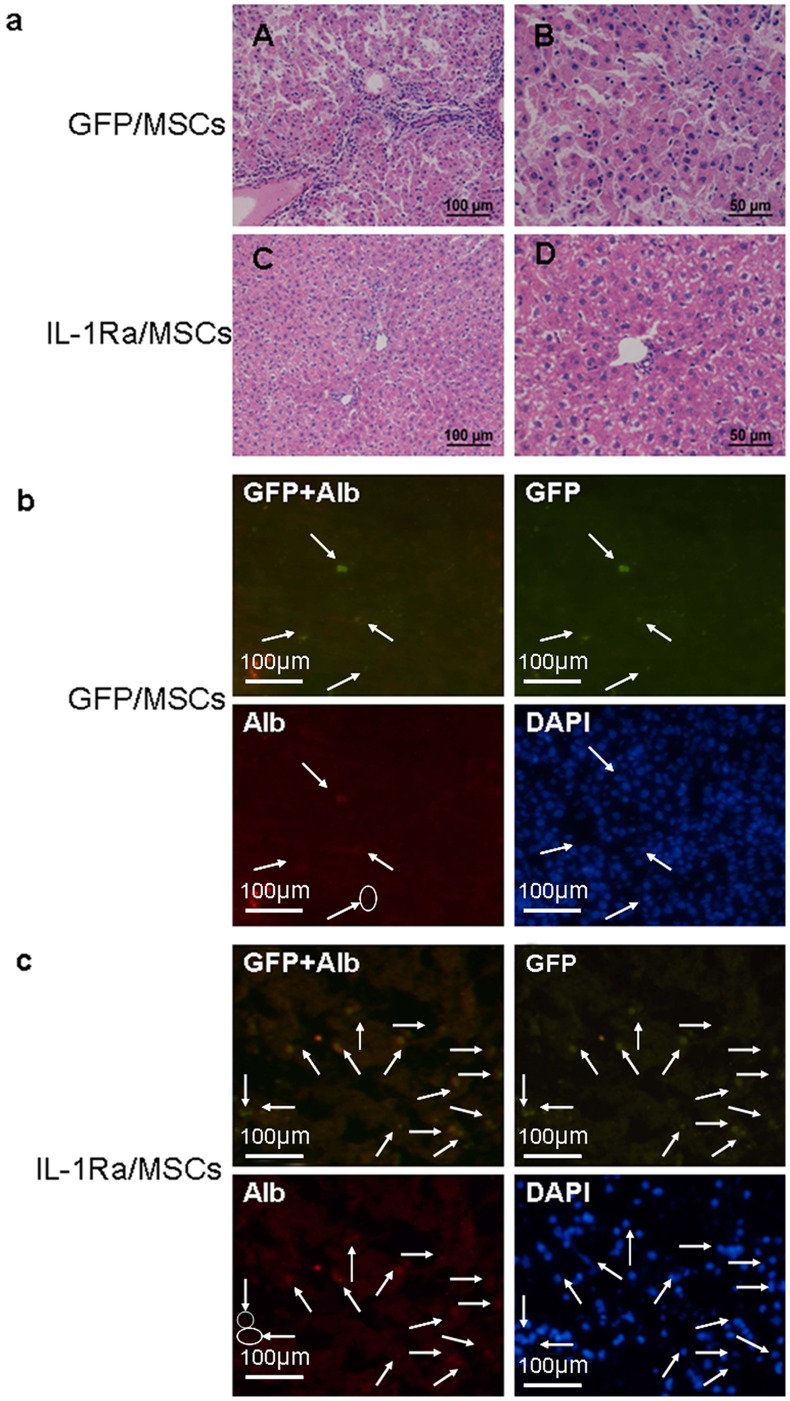
The incorporation of MSCs into injured livers and differentiation of hepatic MSCs in vivo. (a, b) Expression of albumin in GFP-labeled MSCs engrafted in liver tissues, detected by immunofluorescence histochemistry of tissues from IL-1Ra/MSC and GFP/MSC rats. A circle refers to no albumin expression in GFP-labeled MSCs in IL-1Ra/MSC and GFP/MSC rats. (c) Histological appearance of the liver 4 weeks after cell perfusion. Apparent hepatocyte degeneration and increased infiltration of mononuclear cells were detected in the green fluorescent protein/mesenchymal stem cell (GFP/MSC) rats (A, B), but not in the IL-1Ra/MSC rats (C, D). Original magnification ×200 (A, C) ×400 (B, D). Original magnification ×400. Alb, albumin; DAPI, 4′,6-diamidino-2-phenylindole.

In order to determine whether MSCs over-expressing IL-1Ra can be incorporated into liver tissues and differentiate into hepatocytes, rats with FHF used in the survival study were killed 4 weeks after cell transplantation, and the GFP-positive cells were quantified. Cryostat sections were examined for GFP expression and localization with a fluorescence microscope. Minimal expression of GFP was seen in GFP/MSC and IL-1Ra/MSC rats on day 5 after cell transplantation. However, 4 weeks after cell transplantation, the number of GFP-positive cells had increased to approximately 8–14 cells/field in IL-1Ra/MSC rats (Figure 6b), while GFP/MSC rats had only 2–5 GFP-positive cells/field (Figure 6a). The frequency of appearance of the GFP marker was significantly higher in IL-1Ra/MSC rats (12.2±2.7%) than in GFP/MSC rats (2.2±1.3%; n = 5, *P*<0.05). GFP expression was not observed in the livers of PS and LV-IL-1Ra rats.

We then determined the degree of hepatic differentiation by graft MSCs using double-fluorescent immunohistochemistry to detect the expression of albumin (red) in GFP-positive (green) cells. Among the GFP-labeled cells, the percentages of double-positive cells were approximately 38.5±3.7% and 34.5±3.1% in IL-Ra/MSC and GFP/MSCs rats, respectively (*P*>0.05), suggesting that the resident MSCs were producing albumin and had differentiated into hepatocyte-like cells in vivo (Figures 6a and 6b).

Notably, conspicuous infiltration of mononuclear cells was detected in the GFP/MSC rats four weeks after cell transplantation but not in the IL-1Ra/MSC rats (Figure 6c).

## Discussion

We have shown that delivering IL-1Ra-expressing MSCs into FHF rats leads to the attenuation of hepatic inflammatory responses, the regeneration of liver cells, the suppression of apoptosis in hepatocytes, and the migration of MSCs into injured livers; these responses result in significant improvements in liver function and survival.

Our previous studies have indicated that human MSCs from different sources are able to differentiate into functional hepatocyte-like cells and, hence, may serve as a tool for tissue engineering and cell therapy for hepatic tissues in vitro and in vivo [18], [19]. However, fulminant hepatic failure is characterized by massive hepatocyte necrosis and inflammation. Endotoxin and inflammatory cytokines are important mediators in acute liver failure, and IL-1 and TNF-α are involved in its pathogenesis [20]. Recent studies reported that these inflammatory cytokines of the acute-phase response in the liver are cytotoxic for both liver regeneration and the hepatic differentiation of engrafted stem cells [21]. In this study, we found that engrafted AF-MSCs can differentiate into hepatocyte-like cells in failing liver tissue. However, the 21-day survival rate in GFP/MSC rats was only 22.2%, while IL-1Ra/MSC rats had a 21-day survival rate of 69.2%. On the basis of these findings, it appears likely that prevention of the inflammatory effects of pro-inflammatory cytokines such as IL-1 are critical for preventing tissue and engrafted-cell damage and increasing survival rates in early phases of hepatic failure.

In a recent publication, researchers reported that IL-1Ra is a naturally occurring anti-inflammatory protein; it competitively blocks the binding of IL-1α and IL-1β to type IL-1 receptors, but exerts no agonist activity [12]. Normally, enough interleukin-1–receptor antagonist is produced to hold IL-1–mediated inflammation at bay. IL-1Ra is also produced by hepatocytes, primarily during inflammatory conditions associated with FHF [21]. In cases of runaway inflammation, there is an insufficient amount of IL-1Ra to control the activity of interleukin-1 [12]. And Gramantieri reported that an imbalance between IL-1β and IL-1Ra at the tissue level may contribute to the pathogenesis and activation of chronic hepatitis C [22]. Hence, the administration of exogenous IL-1Ra or other agents that reduce the effects of IL-1 should ameliorate inflammatory diseases [12]. Unfortunately, exogenous IL-1Ra is rapidly cleared from the circulation by the liver and thus has a short biological half-life. It is therefore almost impossible to sustain a constant high level of exogenous IL-1Ra in the circulation, even by using repeated injections of IL-1Ra protein at short intervals. However, genetically modified MSCs can downregulate local and systemic pro-inflammatory responses by secreting IL-1 receptor antagonists and can up-regulate anti-inflammatory cytokines such as IL-10 [21]. Thus, a logical way to overcome the problem of rapid clearing of exogenous IL-1Ra is to develop a gene transfer strategy that would allow persistent expression of IL-1Ra protein in vivo. Our study demonstrated that genetically modified mesenchymal stem cells can attenuate local and systemic pro-inflammatory responses. These cells downregulate key acute-phase mediators, including IL-1β, IL-6, and TNF-α, in rats with FHF by over-expressing IL-1Ra. There were no differences in ALT levels between the 4 groups on days 11 and 14, possibly due to compensatory repair processes or stimulation by other endogenous growth factors (e.g. HGF) produced in animals in the PS and GFP/MSCs groups. However, the ALT levels were significantly lower in IL-1Ra/MSC rats than in the PS and GFP/MSC rats at days 5 and 7 after cell transplantation. All of the rats with high ALT levels in the PS and GFP/MSC groups died in the first week due to liver failure.

Our recent studies have shown that hepatocyte regeneration and hepatic differentiation were insufficient in mesenchymal stem cells under hepatic injury conditions [19]. In addition to downregulating hepatic inflammatory responses, IL-1Ra strongly promotes hepatocyte proliferation [23]. Our study has also demonstrated that genetically modified AF-MSCs were transfused into the injured liver and contributed to liver regeneration as shown by BrdU incorporation. In our study, the positive staining rates for BrdU in hepatocytes were also significantly higher in IL-1Ra/MSC rats than in GFP/MSC and LV-IL-1Ra rats 3 days after cell perfusion. And concomitant with this suppression of inflammatory responses, IL-1Ra/MSCs can differentiate into hepatocyte-like cells as previously described in our vitro study [18], [19]. IL-1Ra/MSCs maintained stable hepatic differentiation in vitro. However, in vivo results of our recent study showed IL-1Ra/MSCs possessed a higher hepatic differentiation potential than GFP/MSCs in failing liver tissue. IL-1Ra may thus be capable of enhancing both the incorporation of MSCs into injured livers and the hepatocyte differentiation capability of MSCs by attenuating intrahepatic inflammatory responses.

Siegel also reported that AF-MSCs were being used as a new tool to study human genetic diseases and are suitable for transfection with exogenous genes [24]. Our study design was based on the hypothesis that genetically modified MSCs will stimulate proliferation of hepatocytes and suppress inflammation by over-expressing IL-1Ra and that MSCs will differentiate into hepatocytes after being transfused into failing liver tissues.

In the present study, we first expanded AF-MSCs over-expressing IL-1Ra ex vivo and then transfused these stem cells into the portal vein of rats with FHF. IL-1Ra was shown to promote proliferation and suppress hepatocyte apoptosis on day 5 after cell transplantation. Immunohistochemical analyses showed that IL-1Ra/MSCs have potent anti-apoptotic and mitogenic effects on hepatocytes. Studies have also demonstrated that IL-1Ra functions as a negative regulator of cell senescence through inhibition of IL-1 signaling and prevents apoptosis in ex vivo expansion of human limbal epithelial cells [14].

Our findings also indicated that transfusion of IL-1Ra/MSCs into rats with FHF significantly improved liver function and reduced hepatocellular damage, ultimately improving the 21-day survival rate. Other studies have shown that transfused MSCs were in the sinusoid for the first week, migrated into the liver parenchyma 1 week after engraftment, and further differentiated into hepatocyte-like phenotypes in two weeks [25]. Our results showed that IL-1Ra enhanced the incorporation of MSCs into injured livers by attenuating intrahepatic inflammatory responses, ultimately leading to a significant improvement in the survival rate of rats through enhanced liver cell proliferation and decreased cell death.

Although our previous study showed that BM-MSCs represent a promising source of autologous cells for cell therapies related to liver failure [19], present therapeutic efficacy was limited to some extent. In clinical trials, BM-MSCs have had beneficial effects on patients with liver disease. However, efficient delivery of cells to target organs is critical to improving their effectiveness. A previous study [26]–[27] demonstrated that fluorescence-labeled stem cells perfused into the tail veins were observed primarily in the liver, lung, and spleen, with little or no accumulation in the other organs. A similar study [27]–[28] recently demonstrated that when fluorescently-labelled MSCs were infused intra-portally into carbon-tetrachloride injured mice, the MSCs bound preferentially to injured liver. These observations may be applicable to the population we studied, although further studies are required to confirm this. We would like to address this issue in our future studies. A recent study showed that AF-MSCs can be used as new tools to investigate the molecular and cell biological consequences of various human diseases [24]. In gene therapy studies, human AF-MSCs were genetically modified with a therapeutic gene; the cells retained a high transduction efficiency when mediated by adenovirus vectors and reserved the ability to differentiate into different lineages [26]–[28]. We have shown here that AF-MSCs can be efficiently infected by lentiviral vectors and retain a high hepatic differentiation potential.

The major risks to be considered in research with HIV-1 based lentivirus vectors are the potential for generation of replication-competent lentivirus (RCL) and the potential for oncogenesis. IL-1Ra/MSCs possess the same proliferation and self-renewal capacity as AF-MSCs. These results demonstrate that modifications to the lentiviral vectors optimized the vector as a tool and conferred superior biosafety for the treatment of diverse terminal liver diseases. In this context, it should be noted that our single-injection gene delivery system using a lentiviral vector was proven to be a safe approach, and no tumors or other lesions were observed in the relevant organs.

In summary, our studies showed that IL-1Ra–expressing AF-MSCs participate in the suppression of local inflammatory responses in the liver and systemic immuno-inflammatory responses. IL-1Ra expressed by MSCs not only protects cell grafts from the serum of patients with FHF injury, but also accelerates hepatocyte and MSC graft regeneration and suppresses apoptosis in hepatocytes after cell-perfusion in FHF models. In addition, IL-1Ra promotes MSC migration and incorporation into liver tissue. These MSC grafts subsequently differentiate into hepatocyte-like cells and are instrumental in increasing the survival rate of rats with FHF. This approach to managing the uncontrolled hepatic immunoactivation associated with FHF using MSCs that are genetically-modified to over-express IL-1Ra may offer potentially better outcomes for the treatment of FHF. However, preclinical trials are still needed to determine the safety and efficacy of these cells before they are widely available to patients. Evidence for the inhibition of IL-1 activity by AF-MSCs with IL-1Ra included non-selective immunosuppression and suppression of inflammation. The actions of AF-MSCs expressing IL-1Ra should be further studied in preclinical trials to determine whether such treatment interferes with antiviral immunity in hosts with liver injury from HBV or HCV infections. These cells should also be further tested to determine their biosafety. Such studies should address the issues of insertional mutagenesis and/or oncogenicity in an animal model, as well as inappropriate suppression of inflammation and biodistribution of these cells in non-target tissues. In summary, well-designed preclinical trials should be conducted to explore the clinical applicability of AF-MSCs expressing IL-1Ra.

## Materials and Methods

### Experimental Animals

Syngenic male S-D rats (8–12 weeks of age, weighing approximately 200 g; the Animal Center of Sun Yat-Sen University, Guangzhou, China) were used in all of the experiments in order to exclude any effects of immunologic interference. The animals were kept in the animal facilities at Sun Yat-Sen University, and the experiments were conducted in accordance with the guidelines approved by the China Association of Laboratory Animal Care.

### Preparation of MSCs

Amniotic fluid MSCs were harvested from pregnant Sprague-Dawley rats at gestation day 13±1d as previously described by Hung-Chuan Pan et al [15]. MSCs between the third and sixth passage were used in the experiments. At least 2×10^5^ MSCs were harvested and re-suspended in 0.1 ml phosphate-buffered saline containing 1% bovine serum albumin (Gibco). The cell suspension was incubated with 0.2 µg fluorescein isothiocyanate- or phycoerythrin- conjugated primary antibody (1∶100 dilution), mouse monoclonal anti-rat CD34 (Santa Cruz Biotechnology, Santa Cruz, CA, USA), anti-CD117, anti-CD29 (BD PharMingen, San Diego, California USA), anti-CD44 (Immunotech, Coulter Company, Marseille, France) anti-CD45 and anti-CD86 (eBiosciences, San Diego, CA, USA) for 40 minutes at 4°C. The mouse IgG1 kappa antibody (Catag Laboratories, Burlingame, CA, USA) was used as an isotype control. MSC surface markers were analyzed by fluorescence-activated cell sorter (FACS Calibur, BD biosciences company, San Diego, CA, USA).

### Lentiviral Vectors and Gene Transduction

AF-hMSCs that stably express human IL-1Ra(hIL-1Ra) were established as previously described [16], and lentiviral vector plasmid containing hIL-1Ra-GFP were prepared as previously described [17] (Addgene Inc, Boston, United States ) (Figure S1). AF-MSCs transduced with lentiviral vectors (LV)-GFP were designated as GFP/MSCs, and those transduced with lentiviral vectors (LV)-IL-1Ra were designated as IL-1Ra/MSCs. Approximately 1×10^6^ IL-1Ra/MSCs were cultured for 24 hours, and supernatants were collected at varying time-points up to 14 days. The IL-1Ra supernatant concentrations were determined by enzyme-linked immunosorbent assay(ELISA) using anti-hIL-1Ra monoclonal antibodies (GeneTex, Inc, Irvine, CA, USA).

### Comparison of Proliferation Characteristics between AF-MSCs and IL-1Ra/MSCs in vitro

Morphological characterization of AF-MSCs and IL-1Ra/MSCs compared over 3 weeks are described in Figure S2A. IL-1Ra/MSCs were cultured as previously described by Hung-Chuan Pan et al [15].

#### Growth kinetics

Following the third passage, AF-MSCs and IL-1Ra/MSCs were seeded at 1000 cells/cm^2^ in six-well plates (Corning, USA). Cells were detached by treatment with 0.25% Trypsin-0.02% EDTA and counted with a hemocytometer at days 3, 6, 9, 12, 15, 18, and 21. Dead cells were excluded by Trypan blue staining (Sigma-Aldrich, China). Both of these experiments were performed in triplicate for each point described in Figure S2B.

#### Measurement of octamer-4 mRNA expression in AF-MSCs and IL-1Ra/MSCs by semi-Quantitative RT-PCR

RNA was extracted from 5×10^5^ cells, including AF-MSCs and IL-1Ra/MSCs from passages 3, 6, and 9 using Trizol solution (TIANGEN Biotech Co., LTD., BeiJing, China). Primers used for amplification are described in Table S1 (Table S1). PCR was performed using the following parameters as previously described [18]. Amplified PCR products were separated on a 2% agarose gel by electrophoresis and the bands were visualized by ethidium bromide and photographed with a UVP Imaging System (UVP Company, Upland, CA USA).

### Hepatic Differentiation of AF-MSCs and IL-1Ra/MSCs in vitro

Hepatic differentiation was induced as in our previous study [18]. Briefly, passage 3 AF-MSCs and IL-1Ra/MSCs were plated at 2×10^4^ cells/cm^2^ in 25 cm^2^ flasks and 12-well plates coated with 5 mg/cm^2^ collagen gel type I (Sigma, USA) in basal medium consisting of 60% (v/v) DMEM and 40% (v/v) MCDB-201 (Sigma, USA) supplemented with 2% (v/v) fetal bovine serum, 100 IE/mL penicillin, 100 mg/ml streptomycin, 1 mg/ml linoleic-acid, 0.1 mM L-ascorbic acid, 0.03 mM nicotinamide, 0.25 mM sodium pyruvate, and 1.623 mM glutamine. At confluence, hepatogenic cytokines and growth factors were added sequentially according to the following schedule. Days 0–2: basal medium +10 ng/ml FGF-4; days 3–5: basal medium +20 ng/ml hepatocyte growth factor (HGF); days 6–18: basal medium +20 ng/ml HGF +1× insulin-transferrin-sodium-selenite and 20 mg/l dexamethasone +1 mM trichostatin A (all from Sigma, USA). Differentiation media were changed every 3 days.

### Measurement of Liver-specific Marker Expression in both Differentiated AF-MSCs and IL-1Ra/MSCs Using Semi-Quantitative RT-PCR and Immunofluorescence

Measurement of liver-specific marker(ALB and AFP) mRNA expression in AF-MSCs and IL-1Ra/MSCs by Semi-Quantitative RT-PCR(Figure S3a). RNA was extracted from differentiated AF-MSCs and IL-1Ra/MSCs during hepatic differentiation using Trizol solution (TIANGEN Biotech Co., LTD., BeiJing, China). Primers used for amplification are described in Table S1 (Table S1). PCR was performed using the following parameters as previously described [18]. Amplified PCR products were separated on a 2% agarose gel by electrophoresis and the bands were visualized by ethidium bromide and photographed with a UVP Imaging System (UVP Company, Upland, CA USA).

The presence of liver-specific markers including ALB and AFP in both differentiated AF-MSCs and IL-1Ra/MSCs(Figure S3b) were detected by immunofluorescent staining as in our previous study [18]. Briefly, at different time points in the differentiation process, cells were permeabilized with 0.1% Triton-X 100 (Sigma, USA) for 30 min and blocked with normal goat serum for 30 min at room temperature. The cells were incubated overnight sequentially at 4°C with primary antibodies raised against α-fetoprotein (AFP, 1∶100; mouse, R&D), albumin (ALB, 1∶100; mouse, R&D), and incubated for 1 h at room temperature with secondary antibody (conjugated-CY3 goat anti-mouse1∶100, Santa Cruz Biotechnology, USA). Subsequently, cells were stained with DAPI (1∶10 000, Sigma-Aldrich, ShangHai, China) and observed under a fluorescence microscope (BX51, Olympus, TOKYO, Japan).

### Experimental Design and Animal Groups

Fulminant hepatic failure was induced by an intraperitoneal injection of 1.5 g/kg galactosamine and 200 ug/kg lipopolysaccharides. On day 1 after the induction, rats with FHF were anesthetized with intraperitoneal injections of 6 ml/kg 5% chloral hydrate (Sigma-Aldrich) and 0.4 mg/kg xylazine (Sangon Biotech Co., Ltd, Shanghai ). The rats undergoing FHF induction were divided into four groups according to the material they would receive by transfusion: (i) physiological saline (PS) (ii) GFP/MSCs, (iii) IL-1Ra/MSCs, and (iv) Lentiviral vectors- IL-1Ra(LV/IL-1Ra) as a positive control. One day after FHF induction, either PS, 1×10^6^ MSCs, or 5×10^7^ transducing units(TU)LV-IL-1Ra in a volume of 0.8 ml were transfused into the portal vein of each recipient rat with 28-gauge needle over a period of 5 minutes. Rats were evaluated every 12 hours and killed if they appeared moribund.

### *Survival* Study

Fifteen rats in each group were used for the survival study. Rats that had lived for more than 21 days after transplantation were considered to be survivors.

### Collection of Serum Samples and Hepatic Tissue Specimens

Rats were killed on days 1, 3, 5, 7, 9, 11 and 14 post-transfusion. Twelve hours before killing, a single dose (50 mg/kg) of BrdU (Sigma-Aldrich) was injected intraperitoneally. To detect serum hIL-1Ra levels on days 0, 2, 4, 6, 8, 10, 12 and 14 after transfusion, blood samples were collected from the tail vein 24 h before sacrificing. Liver tissues were excised and processed for further RNA analysis and western blot analysis. The remaining tissue was fixed and processed for histology and immunohistochemistry.

### Measurement of Hepatic Enzyme and Cytokine Levels after Cell Transfusion

The plasma levels of alanine aminotransferase (ALT) and aspartate aminotransferase (AST) after cell transfusion were measured in a biochemistry laboratory (AEROSET, Abbott, USA). The levels of plasma IL-1Ra, IL-6, IL-1β and TNF-α were detected using a commercially available ELISA kit according to the manufacturer’s instructions (IL-1Ra and IL-6, BioSource Corporation, Chantilly, Virginia, USA; IL-1β and TNF, Invitrogen, CA, USA).

### Measurement of hIL-1Ra mRNA Expression in Hepatic Tissues by Semi-Quantitative RT-PCR

Total RNA was isolated using Trizol solution (TIANGEN Biotech Co., LTD., BeiJing, China). Primers used for amplification are described in Table 1. PCR was performed using the following parameters as previously described [18]. Amplified PCR products were separated on a 2% agarose gel by electrophoresis and the bands were visualized by ethidium bromide and photographed with a UVP Imaging System (UVP Company, Upland, CA, USA).

**Table 1:** Primers used for RT-PCR.

Gene	Sequence	Product (bp)
hIL-1Ra	F: 5′-GCCGACCCTCTGGGAGAAAA-3′	356
	R: 5′-GTCTGAGCGGATGAAGGCGA-3′	
GAPDH	F: 5′-CTGCCCCCTCTGCTGATG-3′	170
	R: 5′-TCCACGATACCAAAGTTGTCATG-3′	

Abbreviations: hIL-1Ra, human interleukin-1 receptor antagonist; GAPDH, glyceraldehyde-3-phosphate dehydrogenase.

### Determination of hIL-1Ra Expression in Liver Tissue Using Western Blot Analysis

Hepatic tissues were homogenized, and whole cellular proteins were extracted. Sodium dodecyl sulfate polyacrylamide gel electrophoresis of cellular extracts (50 µg) was performed using 10% acrylamide gels, followed by electrophoretic transfer to polyvinylidene difluoride membranes (Sigma-Aldrich, PeiJing, China). The membranes were then probed with a rabbit anti-hIL-1Ra polyclonal primary antibody (GeneTex, Inc, USA), followed by a horseradish peroxidase-conjugated secondary antibody (Sigma-Aldrich, PeiJing, China). Positive signals were detected using the enhanced chemiluminescence method. Equal loading was assessed by β-actin.

### Measurement of BrdU Incorporation and Apoptosis by Immunohistochemical Assay

Paraffin sections of hepatic tissues were prepared. Bromodeoxyuridine(BrdU) incorporation was determined using a mouse anti-BrdU antibody conjugated to peroxidase (1∶200, R&D Systems, ShangHai, China). Apoptosis was detected by in situ determination of DNA fragmentation using terminal deoxynucleotidyl transferase- mediated 2′-deoxyuridine 5′-triphosphate nick-end labeling (TUNEL) assay. The proliferative index and apoptotic index were quantitatively assessed by calculating the percentage of BrdU-labeled hepatocytes and TUNEL-positive per 1000 hepatocellular nuclei (200-fold magnification), respectively.

### Determination of the Extent of Transfused MSC Retention in Liver

Cryostat sections of livers were prepared and directly estimated for GFP expression and localization using 4′, 6-diamidino-2-phenylindole (1∶10 000, DAPI, Sigma-Aldrich, ShangHai, China), counterstaining, and a fluorescence microscope (BX51, Olympus, TOKYO, Japan). The number of GFP-labeled hepatocytes per 1000 hepatocellular nuclei (200-fold magnification) was also determined. The presence of albumin cryostat sections were detected by immunofluorescent staining as previously described by [6].

### Statistical Analysis

All data were expressed as(Means ± SD). Comparisons involving one independent factor (e.g., treatment) or two independent factors (e.g., treatment and time) were analyzed using one-way and two-way ANOVA, respectively, followed by Bonferroni post-hoc testing. Results from survival experiments were analyzed using the log-rank test and expressed as Kaplan-Meier survival curves. P-values less than 0.05 were considered to be statistically significant. Statistical analysis was performed using SPSS v16.0 software((SPSS, Chicago,IL, USA).

## Supporting Information

Figure S1 **Lentiviral vector hIL-1Ra-GFP. pWPXL-MOD(TG-005), Lentiviral vector plasmid containing hIL-1Ra-GFP (11.821 kp).** The IL-Ra gene is inserted between the Mlu1 and SpeI sites in the lentiviral vector pWPXL-MOD.
(TIF)

Figure S2 **Proliferation characteristics, including morphological characterization (a), Growth kinetics (b), and the expression level of Octamer-4 (Oct-4) mRNA in AF-MSCs and IL-1Ra/MSCs (C).** (a) Morphological characterization of AF-MSCs and IL-1Ra/MSCs. AF-MSCs and IL-1Ra/MSCs with a fibroblastoid, spindle-shaped morphology; there was almost no difference in cell morphology between AF-MSCs and IL-1Ra/MSCs at the 3rd, 6th and 9th passages. Moreover, AF-MSCs and IL-1Ra/MSCs at the 9th passage had no obvious morphological alterations compared to those at the 3rd passage. Phase contrast: magnification ×100 for all figures. (b) At t = 0, both types of MSCs (passage 3) were seeded in six-well plates (1000 cells/cm^2^). Cultures from duplicate wells were harvested every 3 days for 3 weeks. Data are expressed as mean ± SE; n = 3; *P>0.05 compared to IL-1Ra/MSCs on days 12 and 21. There was no difference in cell growth kinetics between AF-MSCs and IL-1Ra/MSCs. (c) From the 3rd to the 9th passage, there was no significant difference in the expression level of Oct-4 mRNA between AF-MSCs and IL-1Ra/MSCs.
(TIF)

Figure S3 **Hepatic differentiation of AF-MSCs and IL-1Ra/MSCs in vitro.** (**a**) The expression levels of specific markers for hepatocyte differentiation (ALB and AFP) were the same in differentiated GFP/MSCs and AF-MSCs. 1: differentiated at day 14; 2: differentiated at day 21. (**b**) IL-1Ra/MSCs and AF-MSCs possess the same hepatocyte differentiation capacity as determined by protein levels using immunofluorescence. Original magnification ×200 for all figures.
(TIF)

Table S1 **Primers for β-actin, Oct-4, AFP, and ALB used for RT-PCR.** Abbreviations: β-actin, Beta-actin; Oct-4, Octamer-4; AFP, α-fetoprotein; ALB, albumin.
(DOC)
