# Supplementary figures and images for: Amniotic-Fluid–Derived Mesenchymal Stem Cells Overexpressing Interleukin-1 Receptor Antagonist Improve Fulminant Hepatic Failure
Source: PLoS One. 2012 Jul 23;7(7):e41392. doi: 10.1371/journal.pone.0041392 (PMC3402415; doi:10.1371/journal.pone.0041392)

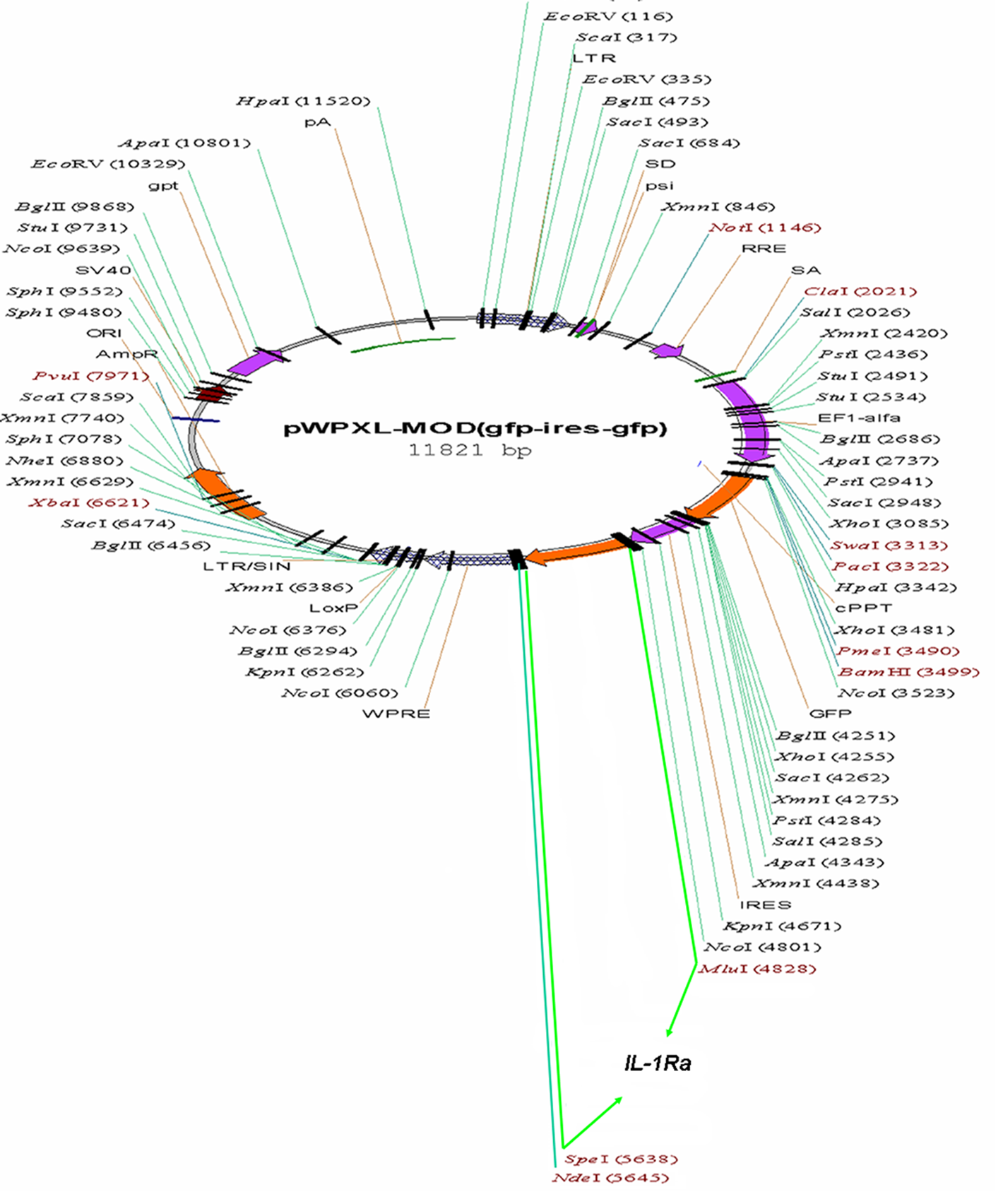

Supplement: Figure S1 — Lentiviral vector hIL-1Ra-GFP. pWPXL-MOD(TG-005), Lentiviral vector plasmid containing hIL-1Ra-GFP (11.821 kp). The IL-Ra gene is inserted between the Mlu1 and SpeI sites in the lentiviral vector pWPXL-MOD. (TIF) [file pone.0041392.s001.tif]

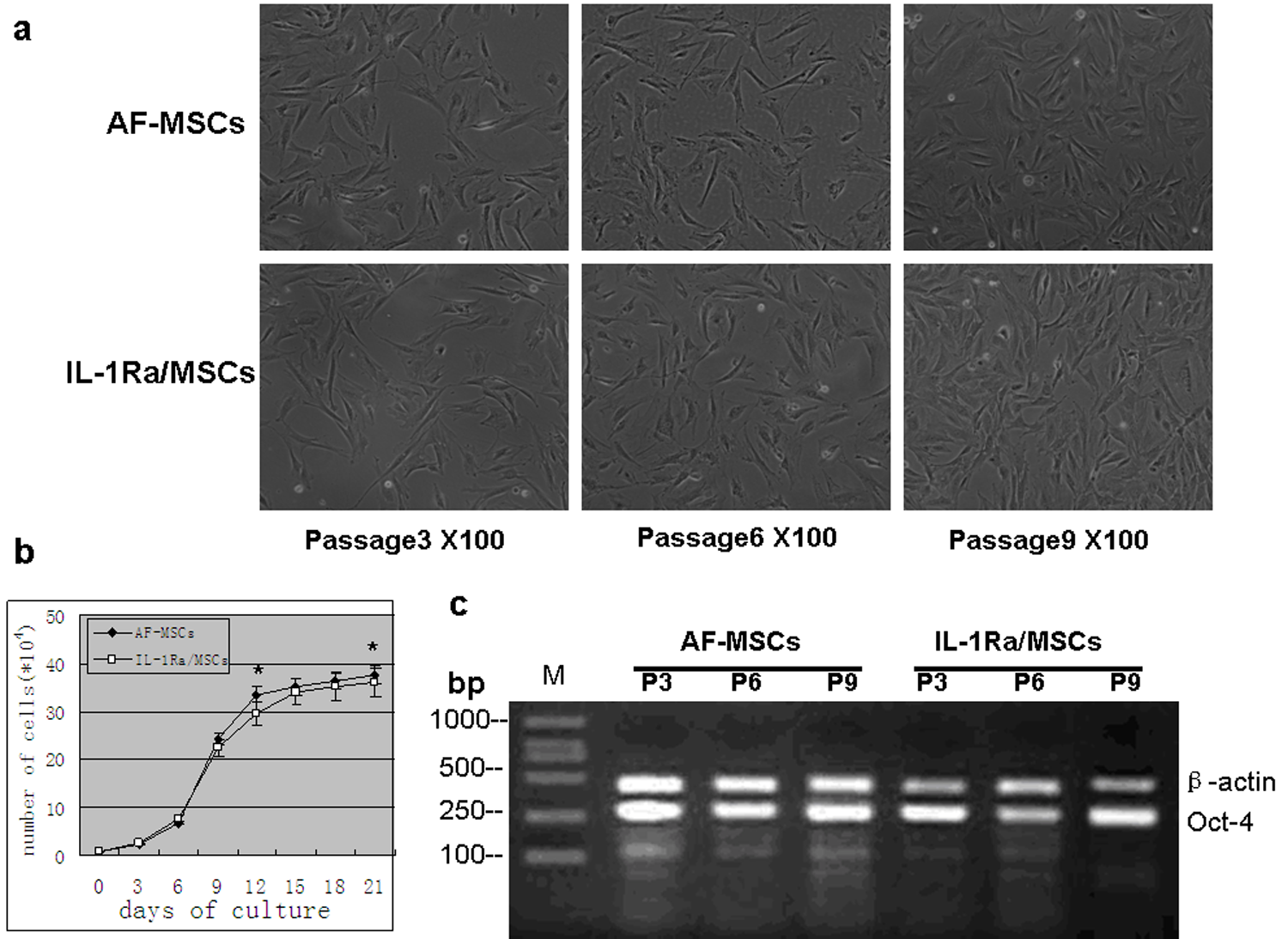

Supplement: Figure S2 — Proliferation characteristics, including morphological characterization (a), Growth kinetics (b), and the expression level of Octamer-4 (Oct-4) mRNA in AF-MSCs and IL-1Ra/MSCs (C). (a) Morphological characterization of AF-MSCs and IL-1Ra/MSCs. AF-MSCs and IL-1Ra/MSCs with a fibroblastoid, spindle-shaped morphology; there was almost no difference in cell morphology between AF-MSCs and IL-1Ra/MSCs at the 3rd, 6th and 9th passages. Moreover, AF-MSCs and IL-1Ra/MSCs at the 9th passage had no obvious morphological alterations compared to those at the 3rd passage. Phase contrast: magnification ×100 for all figures. (b) At t = 0, both types of MSCs (passage 3) were seeded in six-well plates (1000 cells/cm2). Cultures from duplicate wells were harvested every 3 days for 3 weeks. Data are expressed as mean ± SE; n = 3; *P>0.05 compared to IL-1Ra/MSCs on days 12 and 21. There was no difference in cell growth kinetics between AF-MSCs and IL-1Ra/MSCs. (c) From the 3rd to the 9th passage, there was no significant difference in the expression level of Oct-4 mRNA between AF-MSCs and IL-1Ra/MSCs. (TIF) [file pone.0041392.s002.tif]

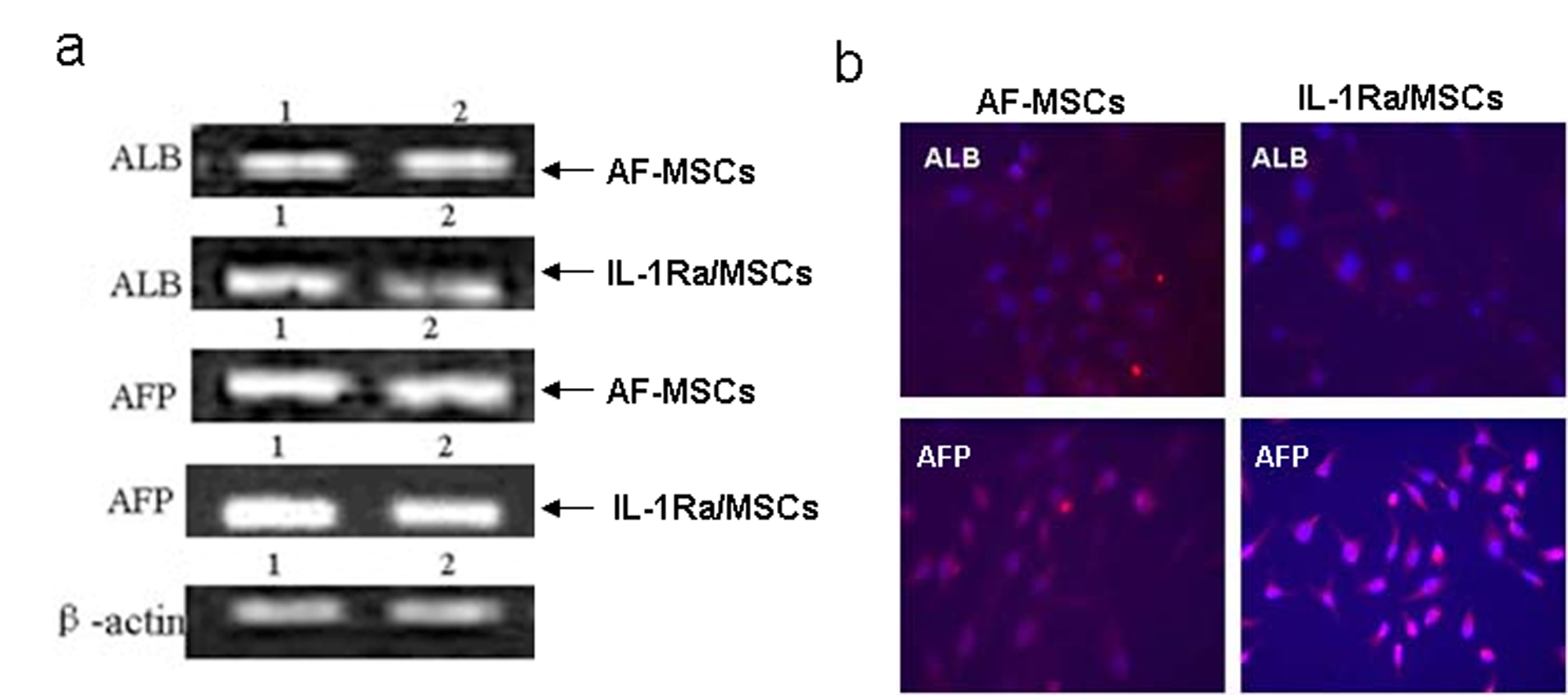

Supplement: Figure S3 — Hepatic differentiation of AF-MSCs and IL-1Ra/MSCs in vitro. (a) The expression levels of specific markers for hepatocyte differentiation (ALB and AFP) were the same in differentiated GFP/MSCs and AF-MSCs. 1: differentiated at day 14; 2: differentiated at day 21. (b) IL-1Ra/MSCs and AF-MSCs possess the same hepatocyte differentiation capacity as determined by protein levels using immunofluorescence. Original magnification ×200 for all figures. (TIF) [file pone.0041392.s003.tif]
