# Supplementary material for: Amniotic-Fluid–Derived Mesenchymal Stem Cells Overexpressing Interleukin-1 Receptor Antagonist Improve Fulminant Hepatic Failure
Source: PLoS One. 2012 Jul 23;7(7):e41392. doi: 10.1371/journal.pone.0041392 (PMC3402415; doi:10.1371/journal.pone.0041392)
Supplement: Table S1 — Primers for β-actin, Oct-4, AFP, and ALB used for RT-PCR. Abbreviations: β-actin, Beta-actin; Oct-4, Octamer-4; AFP, α-fetoprotein; ALB, albumin. (DOC) [file pone.0041392.s004.doc]

Table S1

Primers used for RT-PCR

| Gene | Sequence | Product (bp) |
| --- | --- | --- |
| b-actin | F: 5’-AACACCCCAGCCATGTACGTT-3’ | 481 |
|  | R: 5’-CCACGTCACACTTCATGATGGA-3’ |  |
| Oct-4 | F: 5’-TGAGGGCGAAGCAGGAGT-3’ | 279 |
|  | R: 5’-TCAAAGCGGCAGATGGTC-3’ |  |
| AFP | F: 5’-TGGAATAGCTTCCATATTGGATTC-3’ | 101 |
|  | R: 5’-AAGTGGCTTCTTGAACAAACTGG-3’ |  |
| ALB | F: 5’-TGAGTTTGCAGAAGTTTCCA-3’ | 293 |
|  | R: 5’-CCTTTGCCTCAGCATAGTTT-3’ |  |

Abbreviations: b-actin, Beta-actin; Oct-4, Octamer-4; AFP, a-fetoprotein; ALB, albumin.
